# Anisotropic Behavior of Al1050 through Accumulative Roll Bonding

**DOI:** 10.3390/ma14226910

**Published:** 2021-11-16

**Authors:** Sasan Sattarpanah Karganroudi, Bahman Hatami Nasab, Davood Rahmatabadi, Mina Ahmadi, Mohammad Delshad Gholami, Mehdi Kasaeian-Naeini, Ramin Hashemi, Ahmad Aminzadeh, Hussein Ibrahim

**Affiliations:** 1 Institut Technologique de Maintenance Industrielle, Cégep de Sept-Îles, 175, rue de la Vérendrye, Sept-Îles, QC G4R 5B7, Canada; Ahmad.Aminzadeh@cegepsi.ca (A.A.); Hussein.Ibrahim@itmi.ca (H.I.); 2 School of Mechanical Engineering, Iran University of Science and Technology, Tehran 13114-16846, Iran; bahman1472@gmail.com (B.H.N.); davood.rahmatabadi@yahoo.com (D.R.); mdelshad1372@gmail.com (M.D.G.); m.kasaeian69@gmail.com (M.K.-N.); rhashemi@iust.ac.ir (R.H.); 3 School of Automotive Engineering, Iran University of Science and Technology, Tehran 13114-16846, Iran; Ahmadi.87mina@gmail.com

**Keywords:** accumulative roll bonding, fractography, pole figures, XRD

## Abstract

In this study, Al1050 sheets were fabricated in five passes using the accumulative roll bonding (ARB) technique. For a more accurate and complete investigation, different tests were used, including a uniaxial tensile test. The results show that elongation increases about 50% for the annealed sample, which is 2.5 times that of the fifth pass (20%). A five-fold increase can be seen in tensile strength, which was 50 MPa in the annealed sample and reached 250 MPa at the end of the fifth pass. The annealed sample’s yield stress was 40 MPa, 4.5 times less than 180 MPa after five passes of ARB. Then, to evaluate sample hardness, the Vickers microhardness test was conducted in the samples’ depth direction, which recorded 39 HV for the annealed piece and 68 HV after the last ARB pass. These results show that the hardness increases by 1.8 times after five passes of ARB. In the next step, by conducting fractography tests after the sample fractures during the tensile test, the fracture’s mechanism and type were identified and explained. Finally, X-ray diffraction (XRD) was employed to produce pole figures of sample texture, and the anisotropy phenomena of the annealed sample and ARBed samples were wholly examined. In this study, with the help of pole figures, the anisotropic behavior after ARB was investigated and analyzed. In each step of the process, observing the samples’ texture states and the anisotropy magnificent was possible. According to the results, normal anisotropy of 0.6 in the annealed sample and 1.8 achieved after the fifth pass of ARB indicates that ARB leads to an increase in anisotropy.

## 1. Introduction

Due to growing inquiries from various industries in specialized fields for different materials with unique properties, researchers have recently paid more attention to research in the area of fine-grained materials [1]. Various industries widely employ such materials, presenting high strength and fracture toughness, acceptable ductility, and corrosion resistance [2]. One of the newest methods employed for producing ultrafine grained (UFGed) materials is the severe plastic deformation (SPD) method [3,4,5]. Since the application of UFGed materials is continuously increasing, studying the SPD method by which these materials are fabricated is essential. The advantages of the SPD method include applying severe plastic strains without inducing changes in the appearance dimensions, altering the grain size even to a nanoscale, improving mechanical properties, and finally, performing the process with simple, ordinary, and inexpensive devices and molds [3,6]. The accumulative roll bonding (ARB) process is among the most practical and lowest cost methods for producing UFGed sheets and composites [4,7]. This method involves performing several continuous functions including sheet cutting, degreasing and brushing to clean the sheet surfaces, bonding the sheets by a normal rolling process, and repeating the above steps [8]. With this low cost, convenient method, it is possible to add different reinforcements while efficiently producing sheets or layered composites. Saito was the first to introduce the process, in 1998, as a new SPD method for achieving a UFGed structure in sheet metals [4,7]. Since then, researchers have tried to understand the process better and have started to develop it by investigating the effect of the involved ARB parameters, conducting hot ARB and applying it on various metals, using different reinforcement particles to produce multilayered composites, and assessing mechanical and microstructural features, texture, and their effects on the ARB process [9,10,11,12,13]. One of the critical issues in ARB and all rolling-based processes is studying the anisotropy and texture, which are essential properties of materials and significantly impact the process results [14,15]. Investigating the ARB process and ARBed samples’ anisotropy behaviors is highly recommended to improve the forming processes and achieve parts with better mechanical properties. Analyzing the texture of the materials, especially those that undergo severe plastic deformation processes or are annealed, helps to better understand the microstructural and mechanical evolutions of the output products. Texture analysis plays a crucial role in controlling the ARBed sheets and metals [16]. During the ARB process, the strain gradient arises through the thickness and leads to microstructure and heterogeneous texture evolution [17]. Therefore, the anisotropy of ARBed sheets and their improved mechanical properties have their own origins in the modified textures, microstructural evolutions, and heterogeneities [17,18,19]. Since the texture of ARBed sample is distinctly different from other rolling process results, it is essential to study texture, the orientation of precipitates, and the mechanical and microstructural properties of the sheets after ARB [20]. Some limited studies on the effects of anisotropy on ARBed sheets are presented below.

In 2010, Beausir et al. observed that the anisotropic behavior of samples prepared in a 45-degree rolling direction was different from those in either rolling directions or perpendicular to them. They attributed this phenomenon to the differences in elongations [21].

Duan et al. investigated the effects of ARB on the recrystallization behavior of Fe/Ni ARBed composite. They observed that although considerable modification of the Fe layer was obvious, there were no sensible changes in the texture of Ni layers. A reduction in planar anisotropy was also seen and attributed to the overall balance of the texture components [22]. Karimi et al. studied the plastic anisotropy and texture modification of Ti/Sic multilayered composite after ARB. They concluded that by undergoing the ARB process, there was significant magnitude of anisotropy. They also found that the normal anisotropy of annealed-ARBed sheets was much higher than that of raw materials [15]. Raei et al. investigated the textural evolution of AA1100 Al alloy processed by ARB. Their results showed that the texture of the ARBed samples was almost the same in each ARB cycle, and the difference was only in the texture intensity [16]. Using the tensile test, electron backscatter diffraction (EBSD), and neutron diffraction, Walid Haliba et al. [18] studied the texture and microstructure of Mg/Al ARBed composite. They found that refined microstructure and equiaxed grains resulted due to the ARB process. The microhardness of the samples also increased as the number of the ARB cycles increased. Tensile and yield strength increased between the first and the third ARB pass because of strain hardening and grain modification. However, their values decreased slightly after the remaining two passes due to cracks.

According to previous studies and the above information [14,15,16,17,18,19,20,21], investigating the anisotropy of the elements produced by deformation processes, such as ARB and other rolling fabrication methods, is of great importance. Therefore, exhaustive macroscopic and microscopic investigations of the anisotropy phenomenon of the rolled components is critical for further applications of the ARB method in industry, which has not been done before. The lack of such works has led this study, for the first time, to comprehensively investigate the microscopical and macroscopical anisotropy of ARBed Al1050 sheets, fabricated in five passes, by analyzing the texture and mechanical properties in three rolling directions, and then conducting uniaxial tensile tests and Vickers microhardness tests on the resultant rolled samples. Finally, fractography images are interpreted for a more precise analysis of the pieces and pole figures are also extracted to justify the anisotropy phenomenon.

## 2. Materials and Methods

### 2.1. Material and ARB Process

Al1050 cut into 100 × 150 × 0.5 mm sheets, with the chemical composition given in Table 1, were utilized in the current investigation. In order to achieve a homogeneous structure, the initial material was fully annealed, and because it belongs to the first series of Al alloys, which are pure Al, it showed a ductile fracture. To elevate the sheets’ mechanical and metallurgical properties, they were kept in a thermal furnace at 365 °C for the exact time of 75 min, and then cooled at ambient temperature (annealing process).

**Figure 1:**
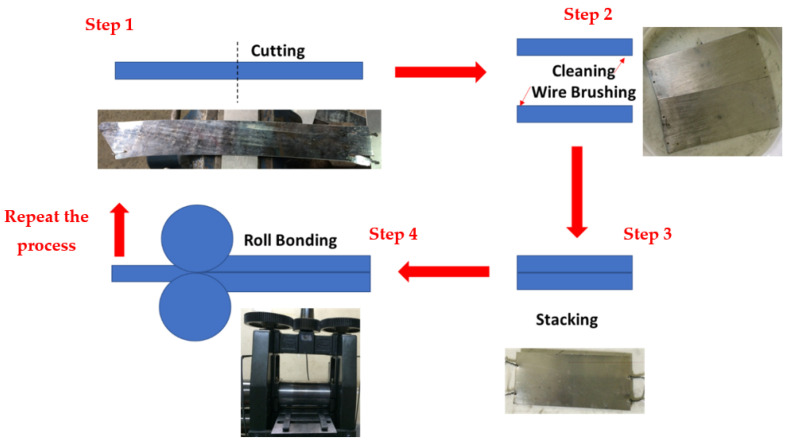
The schematic of the steps involved in the ARB process.

According to Figure 1, the main steps of the ARB process included surface preparation, stacking the layers, applying thickness reduction by rolling, halving the samples, and repeating these steps. The most crucial step in the ARB process is surface preparation, which is usually done chemically and mechanically to ensure more excellent reliability and quality of the connection between layers. Therefore, samples were kept in an acetone solution for 30 min, and their surfaces were scratched via a circular stainless steel wire brush, which was the chemical and mechanical aspects of the preparation, respectively.

After annealing, sheets were sliced into two parts from the centerline in the rolling direction (which is the longitudinal direction); then, one sheet was put on top of the other and went under the rolling process (this process was repeated in each step). As explained previously, to increase the possibility of adhesion between the layers, their surfaces were cleaned via acetone and scratch brushing. The rolling direction during all 5 ARB passes remained constant and in the longitudinal direction. To prevent the sheets from slipping under the rollers, the sheets were attached and fixed together before the rolling operation was performed. The thickness reduction of each rolling pass was 50%. There were five passes of ARB, and after the last step, the emergence of side cracks indicated that no further steps were possible. Figure 1 illustrates the ARB process. The ARB process was performed at ambient temperature (without heat treatment during passes) using a laboratory roller with a capacity of 20 tons and roll diameter of 110 mm.

### 2.2. Tensile Test

**Figure 2:**
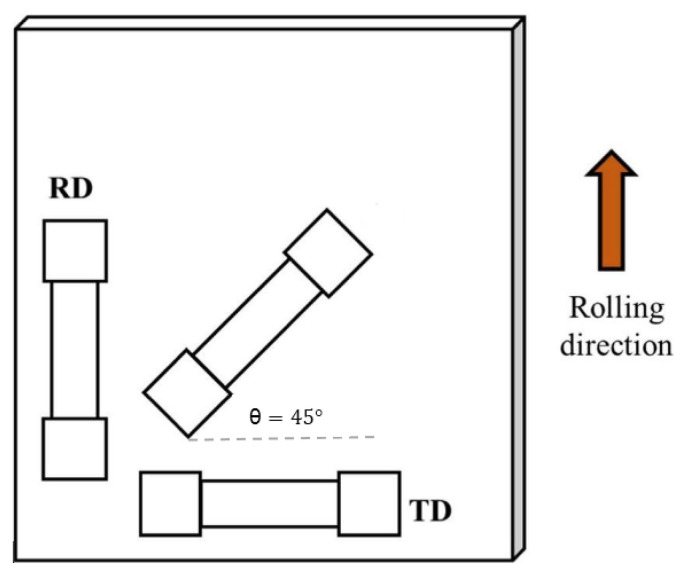
The three rolling directions, according to which the tensile test samples were cut.

To prepare the specimens for the tensile test, they were cut in 3 directions: rolling direction (RD), perpendicular to the rolling direction (TD), and at an angle of 45 degrees so that it was possible to study the anisotropy (Figure 2). The tensile test was performed on 18 samples (6 specimens in each direction) at room temperature and with a strain rate of $\varepsilon =1\times 10^{-3}s^{-1}$.

### 2.3. Anisotropy Evaluation

**Figure 3:**
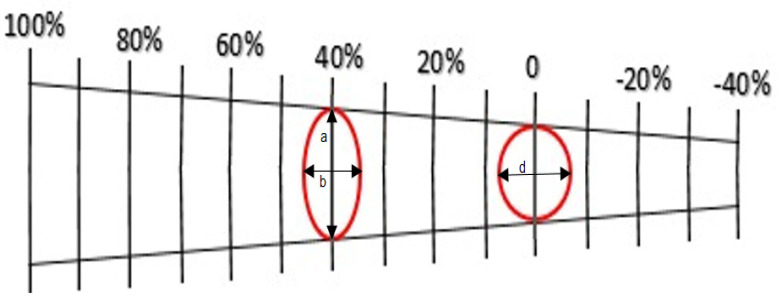
Mylar tape, showing the diameters of d, a, and b.

To calculate the samples’ anisotropy, they were all graded using an electrochemical engraving machine before going under the tensile test. After specimens failed, the ellipses’ diameters close to the fracture point were measured using Mylar tape. (In fact, circles with a diameter of d turn into an ellipse with large and small diameters, a and b) (Figure 3).

Based on the theory of elasticity, engineering strain “e” can be calculated, using Equation (1):

(1) $$e=\frac{\delta }{L_{0}}$$

where δ is the deformation/elongation (a difference between the initial and the final length of the sample after it has been loaded, $L-L_{0}$), and $L_{0}$ is the initial/original length.

For true strain, instead of using the total elongation δ and the original value $L_{0}$, many scientists use all of the values of ***L*** that they have recorded. Dividing each increment ΔL of the distance between the gage marks by the corresponding value of ***L***, the elementary strain will be $\Delta \varepsilon =\frac{\Delta L}{L}$. Adding the successive values of Δε, the true strain will be:

(2) ε=∑Δε=∑(ΔLL)

Which can be written as follows:

(3) ε=∫L0LdLL=lnLL0=ln(1+e)

According to Equations (3)–(8), it was possible to calculate the longitudinal and transverse strains for each sample by measuring the elliptical diameters:

(4) $$e_{Major}=\frac{a-d}{d}\times 100$$

(5) $$e_{Minor}=\frac{b-d}{d}\times 100$$

(6) $$\varepsilon_{Major}=\ln\left(1+e_{Major}\right)=\varepsilon_{l}$$

(7) $$\varepsilon_{Minor}=\ln\left(1+e_{Minor}\right)=\varepsilon_{w}$$

Having $\varepsilon_{l}$ and $\varepsilon_{w}$, $\varepsilon_{t}$ will be:

(8) $$\varepsilon_{t}+\varepsilon_{w}+\varepsilon_{l}=0$$

Then, the values of anisotropy (Lankford coefficient), normal anisotropy, and plane anisotropy **Δr** were calculated as Equations (8)–(10):

(9) $$Rvalue=\frac{\varepsilon_{w}}{\varepsilon_{t}}=\frac{\varepsilon_{w}}{-\varepsilon_{l}-\varepsilon_{w}}\;\;$$

(10) $$r_{m}=\left|\frac{r_{RD}+r_{TD}+2r_{45}}{4}\right|$$

(11) $$\Delta r=\left|\frac{r_{RD}+r_{TD}-2r_{45}}{2}\right|$$

where $\varepsilon_{w}$, $\varepsilon_{l}$ and $\varepsilon_{t}$ are the strains in the direction of the width, length, and thickness, respectively. Moreover, $r_{RD}$ is the anisotropy in the rolling direction and $r_{TD}$ is the anisotropy in the direction that is normal to the rolling direction.

### 2.4. Vickers Microhardness Test

The Vickers microhardness test was performed according to the ASTM-E384 standard. A load of 0.1 kg was applied for 20 s, and then the hardness was measured for each sample at three different points. A sample’s hardness was calculated using the average of the three hardness measurements, and the results were extracted in the form of numbers and diagrams, and then compared.

### 2.5. Fractography

For the fractography test, cross-sections of the fractured samples were examined using scanning electron microscopy (SEM) after the tensile test. X-ray diffraction (XRD) was also employed to measure the texture characteristics of the sheets. For this experiment, 20 × 15 mm samples were prepared from annealed, first pass, and fifth pass samples. The selected plane for X-ray radiation to the pieces was {111}, which finally led to the pole figure images’ results.

## 3. Results and Discussion

### 3.1. Tensile Strength

**Figure 4:**
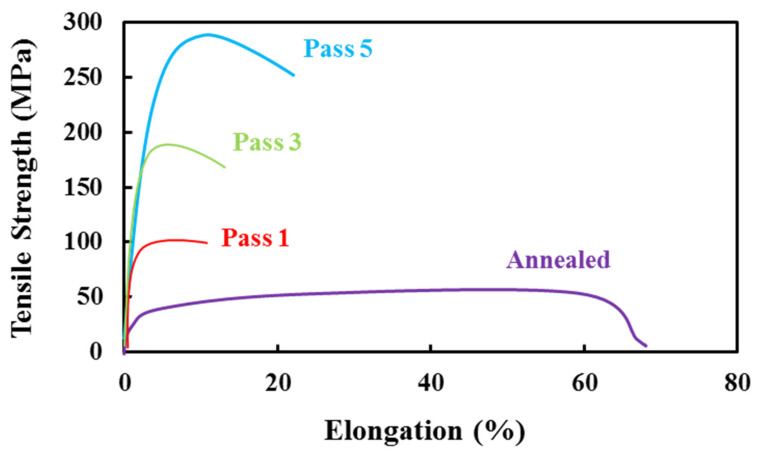
The stress–strain curves of the annealed sample and the 5 ARBed samples in the rolling direction.

**Figure 5:**
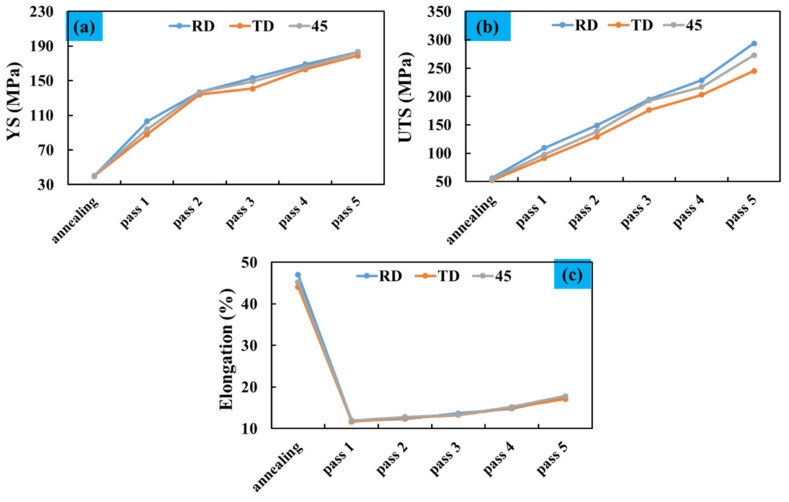
The variations of annealed sample and the 5 ARBed samples in the RD, TD, and 45° rolling directions (**a**) yield stress (**b**) Ultimate Tensile Strength (UTS) (**c**) elongation.

Figure 4 shows the samples’ stress–strain curves obtained from the annealed sheet and processed by five passes of the accumulative rolling process. Samples were cut into a Gauge, length 6 mm, width 4 mm, and thickness 0.5 mm. According to the figure, the ARB samples’ stress–strain curves are different from that of the annealed sample in terms of strength and elongation. This difference is because of an increase in final stress and yield stress after the accumulative rolling process; however, the ability of samples to change their length decreases after the ARB process [7,23,24]. The annealed sample’s elongation is much higher than those of the ARBed samples. For instance, the tensile strength of 56 MPa and elongation of 47% are recorded for the annealed sample, while for the workpiece prepared in the rolling direction and after the third pass, these values are 181 MPa and 13.7%, respectively. This trend is true for all ARBed samples in all three rolling directions (Figure 5).

It is clear from Figure 4 and Figure 5 that, for the samples prepared in the 45-degree rolling direction and the two other rolling directions (RD and TD), the annealed specimen has the highest elongation. After a considerable reduction in elongation, from the initial phase to the first ARB test, it starts to increase by conducting the ARB passes. Ultimate tensile stress and yield stress for samples (annealed and ARBed) in all three rolling directions are the lowest for the annealed sample and increase with the number of passes, until these values are the highest after the fifth cycle. For instance, the Ultimate Tensile Strength (UTS) of the annealed sample, also known as the initial sample in this study, was around 55.8 MPa in the rolling direction and increased to 293.7 MPa after the fifth ARB stage. In the traverse direction, which is normal to the rolling direction, these amounts for the initial and the last phases were 52.2 and 245 MPa, respectively, i.e., an increase of 193 MPa. The same trend is seen for the samples in the 45-degree rolling direction; the increase in tensile strength in this direction is also significant, i.e., an 80% improvement in strength. A continuous increase in tensile strength of the samples from the initial stage to the last stage (fifth cycle of the ARB) can be seen for the samples, but it is more considerable for the RD samples. This phenomenon can be attributed to the fact that grains are elongated in the rolling direction, and cracks need to break the grains’ boundaries in the normal direction. Therefore, the strength in this direction is higher than those in the traverse and 45-degree rolling directions.

For the ARBed specimens, work hardening and grain modification result in differences in the amounts of yield stress and tensile strength. For the first cycles of the ARB process, the dominant phenomenon is strain-hardening, which plays the most crucial role, and therefore the highest increase in UTS occurs in the first pass of ARB. As the number of process cycles increases, due to saturation of the dislocation densities, the strain hardening effect gradually decreases, and the microstructure modification is an essential factor associated with the changes in strength and elongation of the further passes [25,26]. Therefore, due to the fineness of grains, the amount of elongation increases slightly in the final cycle. However, as compared with the annealed sample, its sharp decrease is still noticeable.

As can be seen, the amount of final tensile strength in the fifth pass has suddenly increased as compared with the previous passes, due to the improvement in mechanical properties. In this pass, the material’s fine grains are the main reason for this trend; additionally, fuzzy changes can be another reason that improves the mechanical properties and increases the material’s strength, but its amount is negligible.

Different studies have been conducted on tensile testing of ARBed samples and have shown that, usually, in the accumulative rolling bonding process, the strength of aluminum specimens reaches more than three times that of annealed specimens, and elongation decreases drastically [27,28,29].

### 3.2. Microhardness

**Figure 6:**
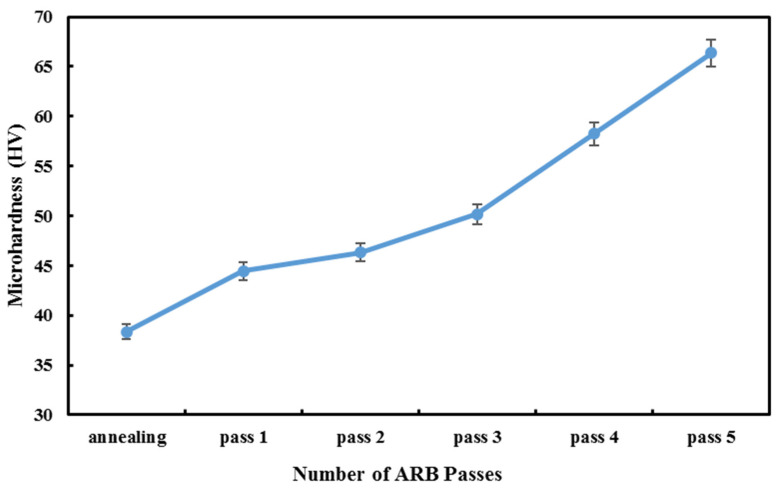
The hardness of ARBed samples and the annealed sample.

The literature confirms that hardness is one of the essential objective functions in the metal forming process in different applications [30,31]. Figure 6 illustrates Vickers microhardness changes for the annealed sample and ARBed samples. It is clear that the annealed specimen has the lowest hardness, and by conducting ARB, it increases as the number of cycles goes up. To minimize the error in hardness calculation, the average of hardness recorded at three points is considered to be the total hardness of each sample.

The sudden increase in the amount of microhardness in the fifth pass as compared with other samples is because of an increase in tensile strength in the fifth pass. In general, after accumulative rolling bonding of aluminum, hardness increases, and this experiment’s results are acceptable [7,23].

### 3.3. Fractography

Due to its face-centered cubic crystal structure (FCC), aluminum shows a soft failure mechanism and has cavities. In this mechanism, failure in the grain boundaries occurs because of low dislocations, and appears in the form of hemispherical holes. These cavities stick together during the tensile test and expand, eventually separating the grains from the surface and creating deep holes at the fracture surface. Finally, a ductile fracture appears as coaxial or hemispherical dimples.

**Figure 7:**
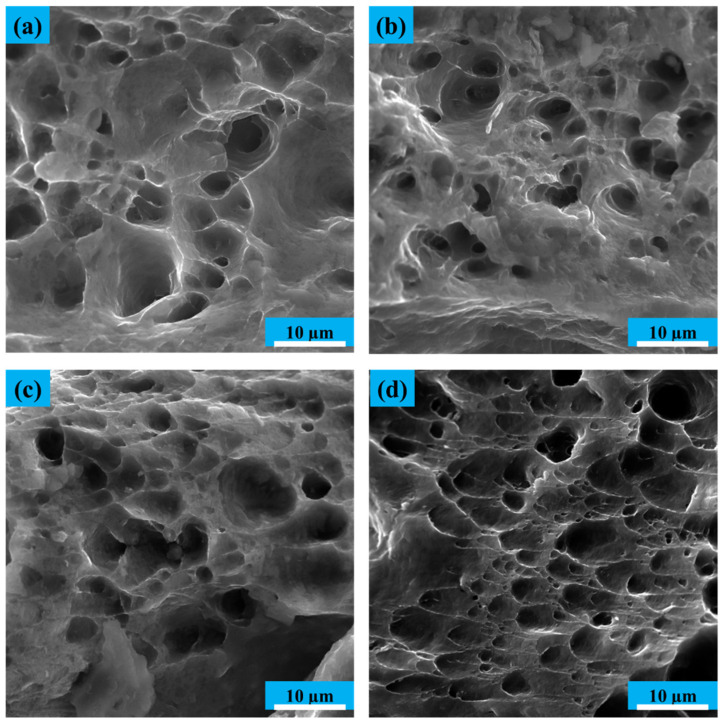
Fracture surfaces of the tensile annealed sample and ARBed samples: (**a**) Annealed sample; (**b**) first ARB pass; (**c**) third ARB pass; (**d**) fifth ARB pass.

In the annealed sample, as shown in Figure 7, the cavities are deep, hemispherical, and in large numbers, indicating a soft failure. As the number of passes of the cumulative rolling process increases, the depth of the holes decreases. Then, the shape of the holes change from hemispherical, and elongated cavities in the shear state emerge. Therefore, the failure mechanism changes from deep cavities and soft failure to become more brittle by increasing the number of accumulative rolling bonding passes. The analysis results of the samples’ fracture images are consistent with other studies’ results and are acceptable.

### 3.4. Texture

In some cases, crystallographic texture, which is the preferred orientation, can be introduced into a material during fabrication. For instance, a sheet texture is often produced when steel sheets are rolled in the manufacturing process. Because of the effects of texture on the material’s properties by inducing anisotropy, measuring the material’s texture seems to be desirable. One typical way of texture measurement is called pole figure, which is a graphical representation of the orientation of objects in space.

**Figure 8:**
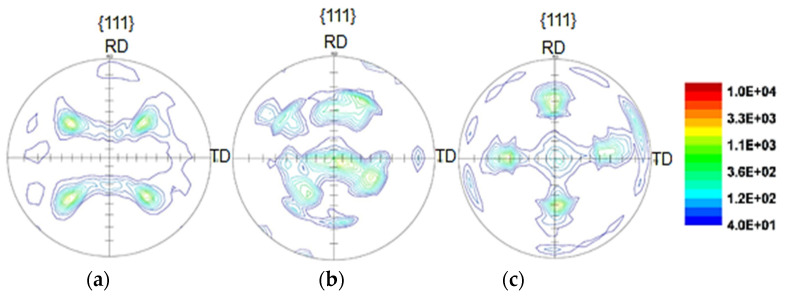
Pole figures of {111} selected plane for: (**a**) Annealed sheet; (**b**) ARBed sample after the first cycle; (**c**) ARBed sample after the fifth cycle.

In this study, for accurate analysis of the anisotropy effect, an XRD test was performed on the samples, and the results are given in the form of pole figure images. Since the texture analysis of the samples contributes to a better understanding of the samples’ textures, there must be a standard XRD analysis of the workpieces before the texture test and production of pole figure images. After selecting the target peak, the spectrum is diffracted at the desired angle by arranging the device with different angles and directions. By comparing the intensity of the peaks, with software help, it is possible to achieve the selected plane’s desired result. The constituent textures of the material for the annealed sheet and the ARBed samples after the first and the fifth pass, are shown in Figure 8, in which the selected plane is {111}.

It is clear from Figure 8a that a relatively strong uniform basal texture is formed parallel to TD for the annealed sample; however, a weaker texture is also seen in the rolling direction. This texture becomes stronger after the first stage of the ARB, but its non-uniformity is evident in this step (Figure 8b). After the fifth pass of the ARB, a stronger basal texture in both rolling directions, RD and TD, is created, which confirms that a more regular and stable structure has been achieved after the final pass of the ARB.

**Figure 9:**
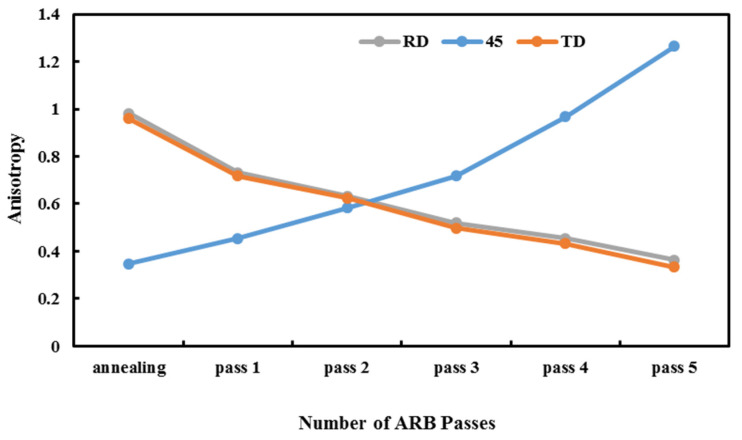
The anisotropy (Lankford coefficient) of the annealed sample and ARBed samples (1–5) in three rolling directions: 45-degree, RD, TD.

As can be seen, the highest distribution intensity is in the fifth pass of the ARBed sample, and the lowest distribution is in the annealing sample. The distribution arrangement of the ARBed sample in the first pass is not uniform; however, this non-uniformity reduces after the fifth ARB cycle, and a more regular arrangement of images is evident in Figure 8c. This regulation is due to improved mechanical properties and modification of grains in the fifth pass as compared with the first pass and annealed sample. By analyzing the above images, it is clear that by increasing the number of ARB passes, the grain’s orientation has changed and become non-uniform. For the annealed sample, the grains are oriented uniformly; however, in the first pass of the ARB, there is a slight rotation in grain orientation, and a mild non-uniformity is observed. However, in the fifth pass, a 45-degree rotation can be observed, which causes a mechanical change in different directions and increases anisotropy as compared with the annealed sample. In the fifth pass, grain non-uniformity and higher distribution intensity of the sheets are entirely distinguished due to the anisotropy in the ARBed plates. Research in this area is very limited, and the results conflict with each other. For example, Karimi et al. produced ARBed samples in up to eight passes and found that, for annealed samples, ARB led to a texture intensity of 1.8, and, in the workpieces which were not annealed, this value was 4.3 [15]. Their results indicated that, although annealing had a significant effect on texture intensity, the ARB process increased the intensity of texture by improving the mechanical properties. Javidikia and Hashemi found that after the SPD process, grain orientation rotated 180 degrees, the distribution intensity increased, and the appearance of the grains became non-uniform, which they attributed to the increase in anisotropy [32]. Pasebani et al. observed that after six passes of the accumulative roll bonding process and according to the pole figure images, the highest value of texture intensity occurred in the third pass of the ARB process; however, in the sixth pass, this amount decreased [33]. In general, using ARB increases texture intensity more than annealing samples. Therefore, the results obtained from the samples’ pole figure images show that this study has more comprehensive results and completes the previous research. In addition, in this study, the rate of the samples’ anisotropy changes is justified by the pole figure images of the workpieces. The Lankford coefficient (also called Lankford value/parameter, R-value, or plastic strain ratio) is a measure of the plastic anisotropy of a rolled sheet metal. By investigating the diagrams in Figure 9, it can be seen that in the samples prepared in the 45-degree rolling direction, the anisotropy increases as the number of passes of the ARB process increase. Still, in the samples prepared in the RD and TD rolling directions, the anisotropy decreases as the number of cycles increases. The reason for such unusual behavior of the 45-degree samples is the difference in elongation as compared with the two other types of ARBed workpieces. For the RD and TD samples, grains are oriented and elongated in just one direction, and after the ARB passes, these grains reach a level that cannot increase more, which can cause a reduction in anisotropy. However, for the 45-degree sample, grains are elongated in both directions, while a strain is applied in just one direction, and grains can grow more than the level of the grains in RD and TD. Therefore, the anisotropy in this direction can exceed that of the RD and TD samples.

**Figure 10:**
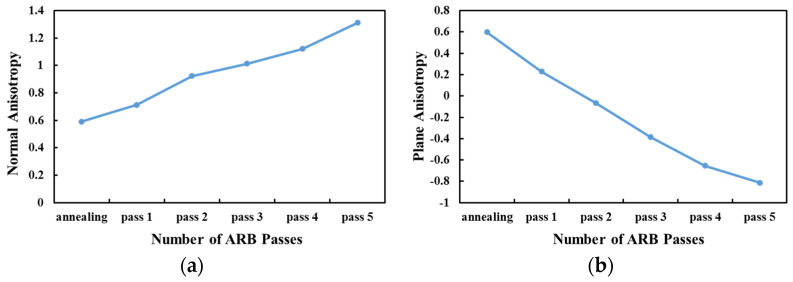
(**a**) Normal anisotropy; (**b**) plane anisotropy of the annealed sample and ARBed samples (1–5).

Normal anisotropy also increases with the number of ARB passes and reaches 1.2 at the end of the fifth pass (Figure 10a). Meanwhile, there is a reverse relation between the plane anisotropy and the number of cycles (Figure 10b).

## 4. Conclusions

In the current study, accumulative roll bonding is an SPD process employed to produce Al1050 sheets in five passes. Investigating the tensile strength, yield stress, elongation, microhardness, fracture behavior, and anisotropy of one annealed and five ARBed samples were the primary objectives of this study. Pole figure images extracted from the XRD test results were used to analyze the anisotropic behavior of the specimen. Tensile test samples, which were also used for fractography, were prepared in three rolling directions: RD, TD, and 45 degrees. For the XRD test, samples were also prepared in exact directions. The study results are as follows:

- For the annealed sample in the rolling direction, the final stress is 56 MPa, and the amount of length change is 47%, while these values for the ARBed sample after the third cycle are 181 MPa and 13.7%, respectively. It shows that the ARB improves the tensile strength of the samples but reduces their elongation. This is the case for the hardness and because of work hardening and high dislocations, the ARBed sample’s hardness is much higher than that of the annealed sample (65 HV for the ARBed and 39 HV for the annealed/initial sample).
- The fracture mechanism of the samples transforms from a ductile fracture with deep cavities in the annealed sample to a brittle failure in the shear state in the ARBed samples.
- Pole figure images show that the grain orientation is more uniform for the annealed sample and starts to be non-uniform by conducting the ARB process. After the first ARB cycle, grain orientation rotates slightly as compared with uniform grains of the annealed sample, and at the end of the fifth ARB pass, there is a 45-degree rotation in grain orientation.
- While there is a direct relationship between the samples’ anisotropy in the 45-degree rolling direction and the number of ARB passes, there is a reverse relation between RD and TD samples’ anisotropic behaviors and the number of cycles. Normal and plane anisotropy increases and decreases, respectively, as the number of ARB passes increase and, in all ARBed samples, are higher than those of the annealed sample.

## Figures and Tables

**Table 1:** Chemical composition (wt.%) of Al1050 used in the current study.

Material	Al	Si	Fe	Mn	Ti	Mg	Zn	Cu
Aluminum 1050	99.421	0.115	0.286	0.029	0.018	0.006	0.016	0.084

## Data Availability

Not applicable.
